# Storage-Induced Fruit Breakdown in *Cryptocarya alba*: Implications for the Conservation of a Keystone Mediterranean Recalcitrant Species

**DOI:** 10.3390/plants14213307

**Published:** 2025-10-29

**Authors:** Viviana Darricarrere, Javier Santa Cruz, Diego Calbucheo, Samuel Valdebenito, Mayra Providell, Mauricio Cisternas, Victoria Muena, Patricia Peñaloza

**Affiliations:** 1 Escuela de Agronomía, Facultad de Ciencias Agronómicas y de los Alimentos, Pontificia Universidad Católica de Valparaíso, Quillota 2260000, Chile; viviana.darricarrere.d@mail.pucv.cl; 2 Escuela de Ciencias Agrícolas y Veterinarias, Universidad Viña del Mar, Viña del Mar 2520000, Chile; santacruz.agr@hotmail.com (J.S.C.); samuelvaldebeni@gmail.com (S.V.); 3 Laboratorio de Ecoinformática, Instituto de Conservación, Biodiversidad y Territorio, Universidad Austral de Chile, Valdivia 5090000, Chile; dgcalbucheo@gmail.com; 4 Escuela de Tecnología Médica, Universidad de Valparaíso, Viña del Mar 2520000, Chile; mlsprovidell@gmail.com; 5 Instituto de Investigaciones Agropecuarias, INIA La Cruz, La Cruz 2280000, Chile; mauricio.cisternas@inia.cl (M.C.); victoria.muena@inia.cl (V.M.)

**Keywords:** flora of Chile, Lauraceae, long-term storage, peumo, plant conservation, sclerophyllous forest

## Abstract

Recalcitrant species are highly sensitive to drought and climate stress, posing urgent challenges for their conservation. Propagation for ex situ management and habitat restoration depends on adequate fruit handling, yet postharvest protocols remain insufficiently examined to support practical implementation. *Cryptocarya alba*, a dominant tree of the Chilean Mediterranean biome, reflects this gap. Despite its ecological relevance and central role in forest planning, the biological basis of its recalcitrant behavior has yet to be fully elucidated, constraining informed decision-making on its propagation. Accordingly, this study examined the progressive breakdown of fruit integrity under two contrasting storage conditions—refrigeration (5 °C) and room temperature (20 °C)—over 150 days, using a multiscale approach combining physical measurements, histology, and scanning electron microscopy. Fruit weight, moisture, pericarp thickness, and cotyledon starch exhibited a significant linear decline over time. The rate was consistently higher at room temperature—except for starch, which showed no quantitative differences across treatments, though the severity of granule alterations was greater. Overall evidence indicates a close association among these variables, suggesting that desiccation and metabolism-driven degradation result in the structural collapse of *C*. *alba* fruits. These findings highlight the need to integrate environmental conditions alongside complementary strategies targeted at physiological regulation, guiding the development of robust, science-based handling protocols to support the species’ conservation.

## 1. Introduction

Mediterranean-type ecosystems are globally recognized for their exceptional biodiversity and high levels of endemism, yet remain among the most threatened biomes due to increasing anthropogenic pressures [1,2,3]. Characterized by a distinctive climate of rainy winters and warm, dry summers, these ecosystems are geographically restricted to five regions aligned across mid-latitudes: the Mediterranean Basin, southwestern Australia, the Cape Floristic Province of South Africa, California, and central Chile [4,5].

In the latter, sclerophyllous forests provide the structural and ecological backbone of the landscape, hosting a wide array of woody species whose reproductive strategies are closely shaped by seasonal climate dynamics. Among them, several produce recalcitrant seeds, particularly those belonging to the families Cardiopteridaceae, Lauraceae, and Myrtaceae [6,7,8]. These seeds maintain elevated metabolic activity upon reaching maturity, rendering them highly vulnerable to progressive dehydration and associated subcellular damage, which rapidly compromises their viability [9,10]. Given these traits, they are especially susceptible to habitat fragmentation and climatic stressors, underscoring the critical need to address their conservation [11,12,13,14].

Handling of propagules is fundamental for preserving genetic resources and enabling plant cultivation for habitat restoration [15,16]. Numerous studies have explored ecological and phylogenetic patterns underlying seed behavior across plant species, advancing our ability to predict storage responses based on shared traits or evolutionary relationships [17,18,19,20]. However, the anatomical and physiological changes associated with viability loss over time remain comparatively underexplored, restricting progress toward evidence-based and effective postharvest practices [21,22].

The Chilean endemic tree commonly known as peumo (*Cryptocarya alba* (Molina) Looser) is one of the dominant and most widely distributed species in sclerophyllous forests [23,24], yet the biological basis of its recalcitrant behavior has been insufficiently studied, hindering propagation efforts [25]. Therefore, to inform better decision-making for their conservation, this study examined the temporal dynamics of structural and metabolic degradation in *C*. *alba* fruits under contrasting storage conditions.

## 2. Results

### 2.1. Fruit Characterization

At collection, fruits had a mean weight of 2.81 ± 0.44 g [1.88–3.67 g], a moisture content of 25 ± 4.0% [23–31%], a length of 1.98 ± 0.17 cm [1.63–2.43 cm] measured along the cotyledonary axis, and a width of 1.67 ± 0.18 cm [1.25–2.22 cm]. Their shape was accurately captured by an ellipse-based equation, which showed a strong fit with the projected area (log-log slope = 0.86, R^2^ = 0.78, *p* < 0.001), that averaged 2.64 ± 0.40 cm^2^ [1.79–3.85 cm^2^].

**Figure 1:**
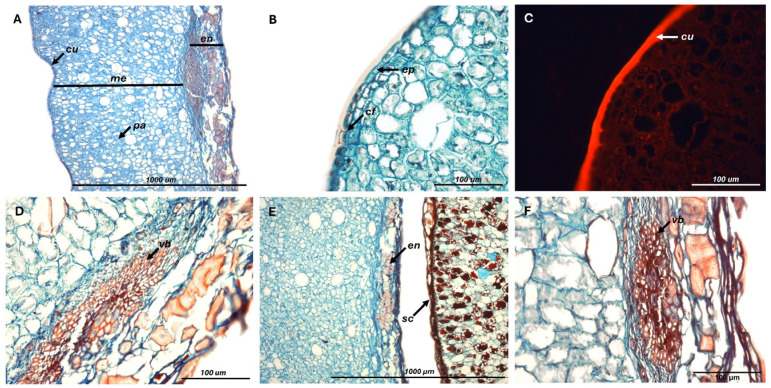
(**A**–**F**) Pericarp structure of fresh *C*. *alba* fruits. Cuticular flanges (*cf*), cuticle (*cu*), endocarp (*en*), epidermal cells (*ep*), mesocarp (*me*), parenchyma cells (*pa*), seed coat (*sc*), vascular bundles (*vb*).

Fruit cuticle measured 9.3 ± 0.9 µm [8.7–10.3 µm], consisting of an outer layer characterized by epicuticular and intracuticular waxes, and an internal one composed of cutin and lignin, which often extended into epidermal cell walls. The mesocarp comprised abundant intercellular spaces surrounded by large parenchymatous cells, irrigated by vascular bundles located near its inner margin. Mesocarp thickness was the most variable among fruit layers (coefficient of variation = 25%), ranging from 795 to 1331 µm (1085 ± 271 µm), and accounting on average for 87% of pericarp thickness. The remaining portion consisted of an endocarp reaching 165 ± 22 µm [146–189 µm], which contributed to tissue rigidity through densely lignified cells oriented in multiple directions (Figure 1). The cotyledons were composed of storage parenchyma densely packed with starch granules (344 ± 59 granules per 1000 µm^2^), which were smooth-surfaced, spherical to slightly oval, and uniformly embedded within the cellular matrix.

### 2.2. Storage-Induced Breakdown

#### 2.2.1. Weight Loss

**Figure 2:**
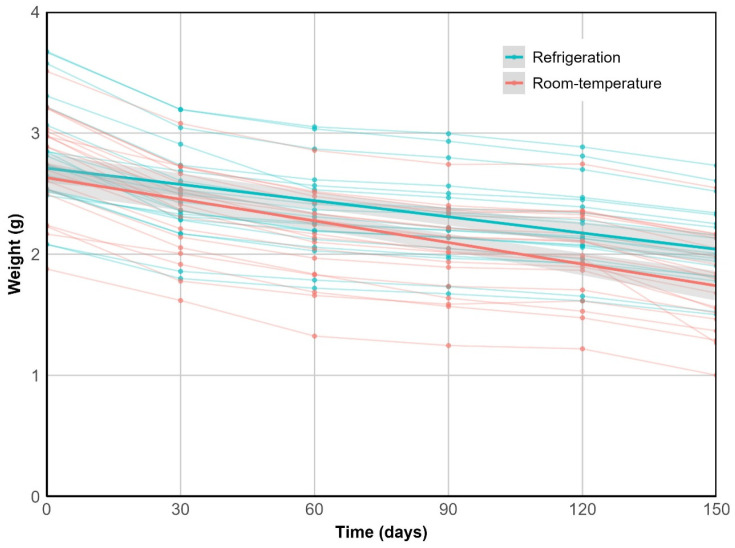
Weight loss over time. Thin lines represent individual fruit weight loss trajectories. Solid lines represent the fixed-effect predictions from the mixed model, summarizing the overall trend for each treatment. This component explained 36% of the variance (R^2^_m_ = 0.36), while the full model—including random effects—accounted for nearly twice (R^2^_c_ = 0.71).

There was a significant decline in fruit weight during storage (*p* < 0.001), with a more pronounced rate under room temperature compared to refrigeration at 5 °C (*p* < 0.05). After 150 days, the latter exhibited 73% of the weight loss observed at 20 °C, where fruits decreased to 1.76 ± 0.39 g. Including fruit-level variability through a mixed model significantly improved data fit (Δ AIC = 131, LRT *p* < 0.001) (Figure 2).

#### 2.2.2. Desiccation

**Figure 3:**
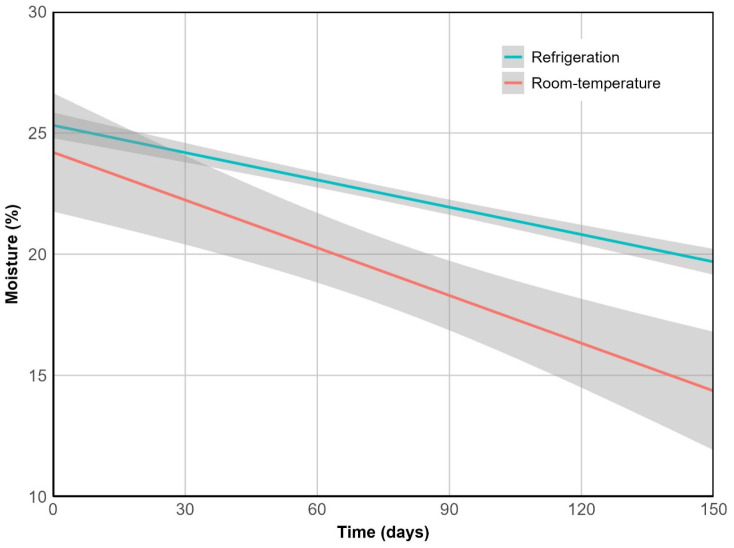
Moisture loss over time. Lines represent predicted values from the linear model with interaction terms, while shaded ribbons indicate the 95% confidence intervals. R^2^ = 0.93, *p* < 0.001.

Moisture content was strongly correlated with weight (ρ = 0.96, *p* < 0.001) and decreased significantly over time under both storage conditions (*p* < 0.001), with a greater decline at 20 °C (*p* < 0.05). This difference was captured by a 1.75-fold steeper slope in the linear model (Figure 3), and by a reduction in measured values from 25% to 14% at room temperature, compared to a final moisture content of 20% under refrigeration.

#### 2.2.3. Tissue Degradation

**Figure 4:**
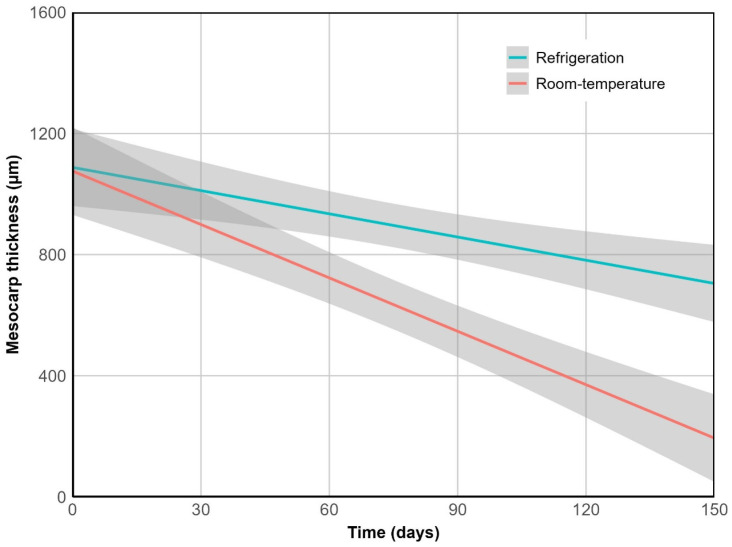
Mesocarp thinning over time. R^2^ = 0.94, *p* < 0.001.

Cuticle thickness remained unchanged over time across storage conditions (*p* = 0.977). However, it showed greater disruption at room temperature, increasing mesocarp exposure to the environment. This was reflected in a significantly higher rate of its thinning under this treatment (*p* < 0.01) (Figure 4). After 150 days, mesocarp thickness decreased by 82% at room temperature (from 1085 µm to 193 µm), compared to only 37% under refrigeration.

This layer emerged as the sole significant predictor of pericarp thinning (*p* < 0.001), exhibiting a strong relationship between both measurements (R^2^ = 0.97, *p* < 0.001). Hence, considering pericarp thickness as a macro-scale indicator of tissue breakdown, a comparable trend was observed: it declined over time (*p* < 0.01) and differed significantly between storage treatments (*p* < 0.01). At room temperature, pericarp thickness decreased by 73% (from 1250 µm to 343 µm), while under refrigeration the reduction was limited to 31%.

**Figure 5:**
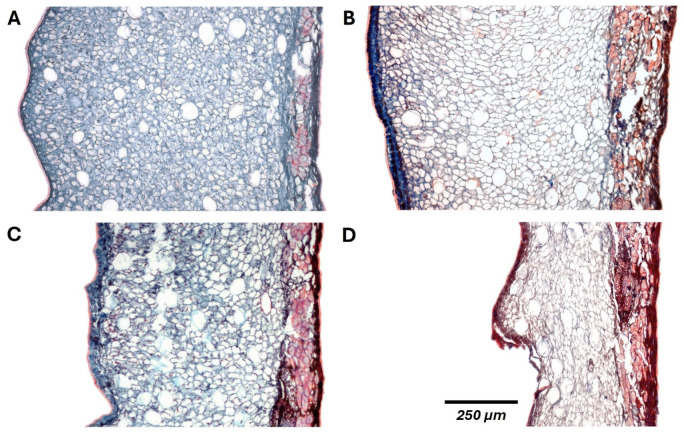
Histological changes in *C. alba* fruits after 30 days (**A**,**B**) and 150 days (**C**,**D**) of storage under refrigeration (**A**,**C**) or room temperature (**B**,**D**).

**Figure 6:**
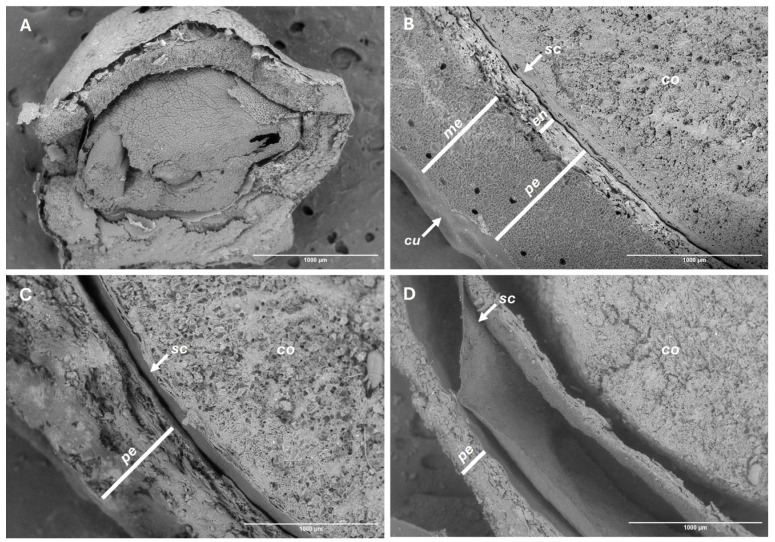
SEM views of fruit integrity. Before storage (**A**,**B**), and after 150 days of storage under refrigeration (**C**) and at room temperature (**D**). Cotyledons (*co*), cuticle (*cu*), endocarp (*en*), mesocarp (*me*), pericarp (*pe*), seed coat (*sc*).

The Spearman test revealed high correlations of both fruit weight and moisture content with mesocarp (ρ ≥ 0.96, *p* < 0.001) and pericarp thickness (ρ ≥ 0.90, *p* < 0.001). Consistently, histological analysis showed a gradual loss of turgor and widespread cell collapse in the mesocarp during storage, accounting for the observed reduction in thickness (Figure 5). These changes were further accompanied by tissue lignification, predominantly in the endocarp, which increased pericarp rigidity and ultimately caused the detachment of both the pericarp from the seed coat and the seed coat from the cotyledons at room temperature, marking irreversible alterations in fruit integrity (Figure 6).

#### 2.2.4. Reserve Depletion

**Figure 7:**
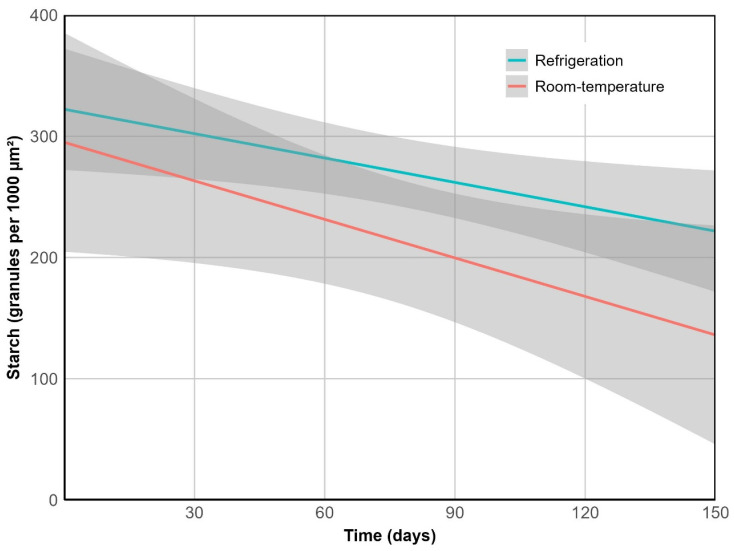
Cotyledon starch depletion over time. R^2^ = 0.68, *p* < 0.01.

**Figure 8:**
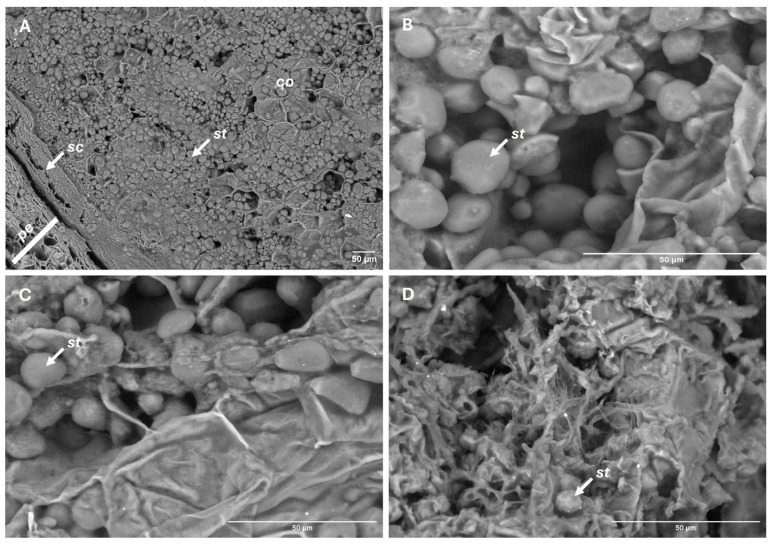
SEM views of cotyledon integrity. Before storage (**A**,**B**), and after 150 days of storage under refrigeration (**C**) and at room temperature (**D**). Cotyledons (*co*), pericarp (*pe*), seed coat (*sc*), starch granules (*st*).

Cotyledon starch granules decreased in abundance at a steady rate over time (*p* < 0.05). Unlike the other variables, this trend did not vary significantly between storage conditions (*p* = 0.370), with an overall reduction of 51% by the end of the experiment (Figure 7). However, marked structural differences were observed. In refrigerated fruits, starch granules retained their morphology and remained embedded within an intact cellular matrix, whereas at room temperature they became irregularly distributed and exhibited surface roughness and fragmentation, consistent with more advanced enzymatic degradation (Figure 8).

## 3. Discussion

Our results reveal a pronounced effect of storage conditions on *C. alba* fruit breakdown. Refrigeration at 5 °C substantially reduced degradation relative to room temperature, aligning with six decades of recommendations advocating cold storage for this species [26,27,28,29,30,31]. By integrating physical, histological, and ultrastructural evidence, our study corroborates and mechanistically underpins that guidance, providing a scientifically grounded basis to inform conservation strategies.

Fruit desiccation, known to begin during maturation [32], proceeds at a constant rate driven by storage temperature, as reflected in changes in weight and moisture content. While earlier works identified the pericarp as a barrier against water loss [33,34,35], our results reveal that this function is progressively undermined by structural breakdown, primarily caused by the loss of mesocarp integrity. Since this tissue consists mainly of water-rich parenchyma cells, storage conditions exert a marked influence on its desiccation, with higher temperatures accelerating the decline of cell turgor and the consequent collapse of the tissue.

By contrast, fruit lignification, reported to decline until ripening [36], intensifies during storage, ultimately enhancing pericarp rigidity and detachment from the seed coat. This loss of structural continuity constitutes a pivotal stage of fruit breakdown, diminishing the protective function of the pericarp through the progressive exposure of the seed to the surrounding atmosphere, a process that was prevented even after 150 days of refrigeration.

Documented differences in pericarp thickness among *C. alba* populations, and variation in fruit shape across provenances and years [32,37,38], are expected to modulate breakdown rates through variable susceptibility to desiccation, driven both by the extent of the protective tissue and by changes in the surface-to-volume relationship. Consequently, additional handling measures should be implemented for fruits with thin pericarps or elongated shapes.

Alongside structural deterioration, seed breakdown is further caused by the persistence of metabolic activity after ripening. In line with previous findings that respiration persists through this stage in *C*. *alba* fruits [32], seeds undergo continuous mobilization of carbon reserves during postharvest, gradually depleting their storage compounds, as reflected in the decline in starch granules recorded in our study. This pattern was partially unaffected by the evaluated storage conditions, reflecting the presence of an endogenous metabolic demand, as also reported for other recalcitrant species [39,40,41]. Therefore, targeted interventions—such as modified atmospheres with reduced oxygen, hormonal regulators, or controlled hydration—should be explored as potential strategies to down-regulate reserve consumption and improve fruit storability.

## 4. Materials and Methods

### 4.1. Plant Material

Fruits of *C. alba* were collected in the Metropolitan Region of Chile (33°24′ S, 70°37′ W) in August 2023, at the Ca3 ripening stage (dark pink fruits) according to the species-specific classification of Valdenegro et al. [32]. They were disinfected using a 1% (*w*/*v*) sodium hypochlorite solution combined with 0.01% (*v*/*v*) Tween^®^ 20 (polysorbate 20) for 15 min, followed by rinsing with distilled water and air-drying on paper towels.

Subsequently, 100 fruits were characterized by evaluating their individual weight, length, width, and projected longitudinal area (2D lateral view), using an analytical balance (±0.001 g precision) and the ImageJ software v1.54 (National Institutes of Health, Bethesda, MD, USA) [42]. The remaining fruits underwent two treatments over a 150-day period: (i) refrigeration at 5.0 ± 0.6 °C (relative humidity 81 ± 7.0%) and (ii) room temperature storage at 20.0 ± 2.8 °C (RH 87 ± 9.3%), both in airtight plastic containers with silica gel sachets. This timeframe was based on the species’ reported propagule viability, and the temperature treatments were chosen to represent typical storage conditions [27,28,33]. Their impact on fruit integrity was evaluated through multiscale analysis performed at 30-day intervals, as detailed in the following sections.

### 4.2. Physical Analysis

Weight was monitored in twenty individually labeled fruits per treatment at each interval, enabling repeated measurements over time. In parallel, moisture content was assessed destructively in four samples of five fruits each by placing them at 130 °C for 2 h in a WiseVen™ WOF-105 drying oven (DAIHAN Scientific, Wonju, Republic of Korea), in accordance with the International Seed Testing Association [43].

### 4.3. Histological Analysis

To assess tissue degradation, three fruits per treatment were periodically fixed in a formalin–acetic acid–alcohol (FAA) solution. The samples were then dehydrated in a graded ethanol series, cleared with xylene, and embedded in Paraplast^®^ paraffin, following standard protocols [44,45,46,47]. Histological sections were obtained using a Thermo Scientific™ HM 325 rotary microtome (Thermo Fisher Scientific, Waltham, MA, USA) set to a thickness of 10 μm. These were stained with safranin O (CI 50240) and fast green FCF (CI 42053) to selectively differentiate lignified and cellulose-rich tissues, respectively [48,49].

The resulting slides were examined under an Olympus^®^ CX31 epifluorescence microscope (Olympus Corporation, Hachioji, Japan) equipped with a U-LH100H6 Hg lamp for safranin excitation (~540–550 nm). Images were captured with a MicroPublisher 3.3 RTV camera (QImaging, Surrey, BC, Canada) and processed using QCapture Pro 5.1 software (QImaging^®^).

**Figure 9:**
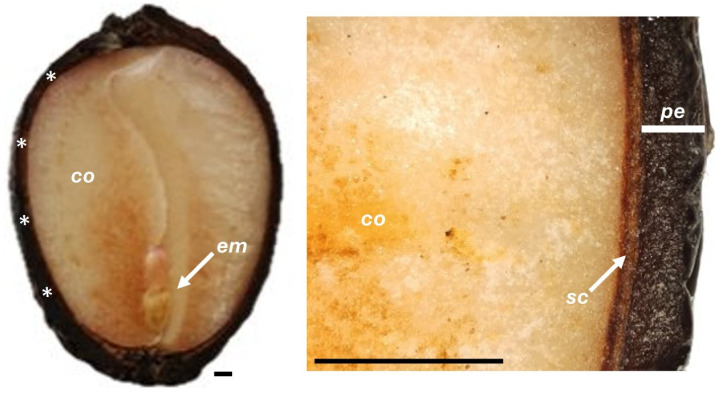
Longitudinal view of fresh *C*. *alba* fruits. Smooth, glossy pericarp (*pe*), enclosing a single seed with a light brown seed coat (*sc*), pale cream cotyledons (*co*) showing slight granular appearance, and a pink embryo (*em*) located at the distal end. Asterisks (∗) indicate the measurement regions of cuticle and pericarp thickness. Scale bars = 1 mm.

The thickness of the cuticle and pericarp layers were measured in four equidistant regions per fruit (Figure 9). Although the latter is theoretically composed of epicarp, mesocarp, and endocarp, the gradual transition between epidermal and parenchymatous mesocarp cells prevented clear distinction of the epicarp. Consequently, both tissues were measured together and reported as mesocarp.

### 4.4. Ultrastructural Analysis

Cotyledon starch reserves were monitored under vacuum using a Hitachi SU3500 scanning electron microscope (SEM) (Hitachi High-Tech Corporation, Tokyo, Japan) equipped with a backscattered electron (BSE) detector. For each treatment, three fruits were analyzed by quantifying starch granules in three randomly selected 11,750 µm^2^ regions from each.

### 4.5. Statistical Analysis

As starch and pericarp layer measurements were taken 3–4 times per fruit, values were averaged to yield a single value. Data normality, homoscedasticity, and residual autocorrelation were evaluated through the Shapiro–Wilk, Breusch–Pagan (or, exclusively for weight, Levene’s test), and Durbin–Watson tests, respectively.

Linear regression models with interaction terms were used to evaluate the effects of treatments on mean fruit moisture, total and layer-specific pericarp thickness, and starch granule count over time. Additional simple models were fitted to explore the relationship between the projected fruit area and that estimated from an ellipse-based Equation (1), as well as between total pericarp thickness and its constituent layers.

(1) $$\mathrm{Estimated}\;\mathrm{area}\;({\mathrm{cm}}^{2})\;=\;\pi \;\times \;(\frac{\mathrm{Length}}{2})\;\times \;(\frac{\mathrm{Width}}{2})$$

Both simple and mixed linear models were tested for weight, incorporating fixed and fruit-level random effects. Model selection was guided by comparisons of Akaike Information Criterion (AIC) and likelihood ratio test (LRT) results. Lastly, Spearman’s rank correlation was used to explore associations among measured parameters.

## 5. Conclusions

Across 150 days of storage, *Cryptocarya alba* fruits exhibited progressive losses of fresh weight and moisture, pericarp degradation, and starch depletion, with consistently faster rates at 20 °C than at 5 °C. Notably, the data revealed differential diagnostic value among breakdown metrics; for instance, mesocarp thinning—rather than endocarp thinning or both jointly—emerged as the main driver of pericarp collapse, whereas changes in cotyledonary starch ultrastructure were more responsive than changes in its abundance. Future work should quantitatively link the most informative metrics to germination and seed viability outcomes. These relationships will refine evidence-based handling protocols to inform the conservation of this keystone recalcitrant species of the Chilean Mediterranean forests.

## Data Availability

All supporting data are presented in the main text. Additional details can be requested from the corresponding author upon reasonable request.

## Abbreviations

The following abbreviations are used in this manuscript:

AIC	Akaike information criterion
BSE	Backscattered electron
FAA	Formalin–acetic acid–alcohol
LRT	Likelihood ratio test
RH	Relative humidity
SEM	Scanning electron microscopy
